# Failure of Passive Immune Transfer in Calves: A Meta-Analysis on the Consequences and Assessment of the Economic Impact

**DOI:** 10.1371/journal.pone.0150452

**Published:** 2016-03-17

**Authors:** Didier Raboisson, Pauline Trillat, Clélia Cahuzac

**Affiliations:** 1 Université de Toulouse, Institut National Polytechnique (INP), Ecole Nationale Vétérinaire de Toulouse (ENVT), UMR 1225, Interaction Hôte Agent Pathogène (IHAP), F-31076 Toulouse, France; 2 INRA, UMR1225, IHAP, F-31076 Toulouse, France; 3 Université de Toulouse, Institut National Polytechnique (INP), Ecole Nationale Vétérinaire de Toulouse (ENVT), F-31076 Toulouse, France; University of British Columbia, CANADA

## Abstract

Low colostrum intake at birth results in the failure of passive transfer (FPT) due to the inadequate ingestion of colostral immunoglobulins (Ig). FPT is associated with an increased risk of mortality and decreased health and longevity. Despite the known management practices associated with low FPT, it remains an important issue in the field. Neither a quantitative analysis of FPT consequences nor an assessment of its total cost are available. To address this point, a meta-analysis on the adjusted associations between FPT and its outcomes was first performed. Then, the total costs of FPT in European systems were calculated using a stochastic method with adjusted values as the input parameters. The adjusted risks (and 95% confidence intervals) for mortality, bovine respiratory disease, diarrhoea and overall morbidity in the case of FPT were 2.12 (1.43–3.13), 1.75 (1.50–2.03), 1.51 (1.05–2.17) and 1.91 (1.63–2.24), respectively. The mean (and 95% prediction interval) total costs per calf with FPT were estimated to be €60 (€10–109) and €80 (€20–139) for dairy and beef, respectively. As a result of the double-step stochastic method, the proposed economic estimation constitutes the first estimate available for FPT. The results are presented in a way that facilitates their use in the field and, with limited effort, combines the cost of each contributor to increase the applicability of the economic assessment to the situations farm-advisors may face. The present economic estimates are also an important tool to evaluate the profitability of measures that aim to improve colostrum intake and FPT prevention.

## Introduction

The failure of the neonatal calf to absorb adequate colostral immunoglobulins (**Ig**) within the first hours of life results in failure of passive transfer (**FPT**). FPT leads to an increased risk of mortality and decreased health and longevity. Depending on how FPT and livestock systems are defined, the prevalence of FPT is reported to reach 20 to 40% of newborn calves [1,2]. Mortality linked to FPT has been reported as ranging from 8 to 25%. Ensuring that calves drink enough colostrum within a few hours of birth is a powerful way to reduce FPT and its associated disorders. The minimal quantity of Ig that the calf needs to absorb to prevent FPT is approximately 150 g [3]. Several practical guidelines to prevent FPT have been proposed for use on farms [4–7]. Management practices that are risk factors for FPT are also well known [1,8,9]. However, FPT remains an important issue on dairy and beef farms. Worldwide, FPT contributes to high and increasing mortality rates of young calves [10]. Because FPT increases the risk of health disorders (mostly bovine respiratory diseases [BRD] and diarrhoea), it also contributes to antimicrobial use and, consequently, to antimicrobial resistance [11].

The consequences of FPT on health are poorly described, and no quantitative overview is available. Moreover, the total cost of FPT has never been reported. A clear overview of the consequences of FTP and an assessment of its total costs would be key to helping farm advisors make decisions. Because FPT is associated with several disorders, even simple economic calculations made at the farms, such as a partial budget analysis, remain difficult and time consuming. Good decision-making requires that the total cost of FPT be accurately determined, with biological and livestock system variability included in the model. The present work aims to estimate the total costs of FPT in European systems using a stochastic method with adjusted values as the input parameters. Such an economic assessment cannot be performed without a preliminary quantification of the adjusted associations between FPT and its outcomes using the changing definitions of FPT and the co-variables from previously published models.

## Materials and Methods

### Meta-analysis

**Fig 1:**
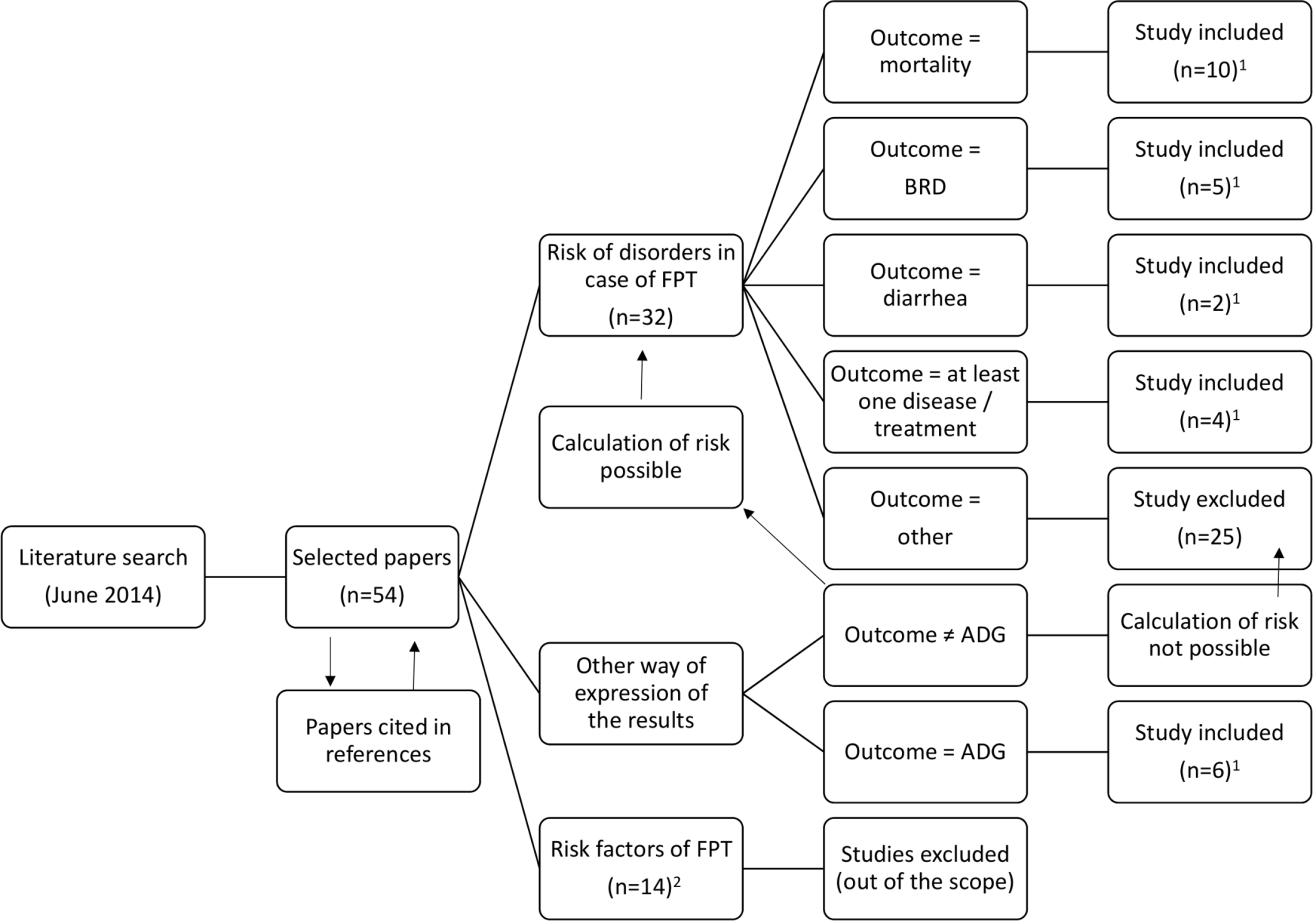
Flowchart on selection of papers. BRD: Bovine Respiratory Diseases; ADG: Average Daily Gain; 1: Some papers included several outcomes at a glance; 2: Some papers were excluded at the previous stage (literature review) based on its title.

A literature search and screening process were conducted using the PubMed, CAB and Google Scholar search engines to create a dataset of papers with the key words “passive immunity”, “IgG”, “immunoglobulins”, “colostrum management”, “colostrum”, and “calf”, separately or in combination. Additional papers referenced by at least 1 of the papers identified in the search were also included. To be included in the dataset, the papers must have examined the risks of various disorders (mortality, all diseases and production changes in calves with or without FPT) and have been peer-reviewed. No other inclusion criteria were used. Exclusion criteria were (i) papers with no quantification of the risk of diseases in case of FPT, since they cannot included in the meta-regression, (ii) definition of PFT that did not fit with the retained one, since they cannot be included in the related co-variables (Table 1) and (iii) outcomes that are not mortality, diarrhoea, BRD or average daily gain (ADG), since other outcome only gather one or two data. Papers published through June 2014 were included (Fig 1).

**Table 1:** Definition of the moderators used in the meta-regression.

Moderator	Classes	Definition
FPT_DIAG1_	1	Threshold^1^: IgG = 3.5–5 g/L or TP = 40 g/L
	2	Threshold: IgG = 8 g/L or TP = 45–50 g/L
	3	Threshold: IgG = 10–12 g/L or TP = 54–55 g/L
	4	Threshold: IgG = 15–24 g/L
FPT_DIAG2_	1	Threshold: IgG = 3.5–5 g/L or TP = 40 g/L
	2	Threshold: IgG = 8–10 g/L or TP = 45–50 g/L
	3	Threshold: IgG = 12 g/L or TP = 54–55 g/L
	4	Threshold: IgG = 15 g/L or TP = 15–24 g/L
FPT_GAP_	0	No gap in IgG or TP to define FPT^2^
	1	A gap in IgG or TP to define FPT^3^
FPT_THRES_	0	FPT is defined relative to all values above or below the threshold^4^
	1	FPT is defined relative to ranges of values above or below the threshold^5^
OUT_TIME_MORT_^6^	1	Duration of observation of the outcomes^7^ = 0 to 60 days of life
	2	Duration of observation of the outcomes = 0 to 90–110 days of life
	3	Duration of observation of the outcomes = 0 to 160–200 days of life
OUT_TIME_RESPI_^8^	1	Duration of observation of the outcomes^7^ = 0 to 1, 3 or 4 months of life
	2	Duration of observation of the outcomes = 0 to 5 or 6 months of life
	3	Duration of observation of the outcomes = 0 to 15 months of life
OUT_MORBIDITY_^9^	1	Models with outcome = “at least one disease or treatment”
	2	Models with outcome = “diarrhoea”
	3	Models with outcome = “BRD”

1: The threshold is used to distinguish between calves with and without FPT. IgG is blood immunoglobulin G, and TP is blood total proteins

2: Calves with FPT have a TP < 50 g/L and calves without FPT have a TP > 50 g/L

3: Calves with FPT have a TP < 45 g/L and calves without FPT have a TP > 55 g/L

4: Calves with FPT have a TP < 45 g/L

5: Calves with FPT have a TP between 40 and 45 g/L

6: For the meta-regression related to mortality only

7: The duration of observation of the outcomes always started at birth and different studies used different endpoints

8: For the 2 meta-regressions related to bovine respiratory disease and at least one other disorder or treatment

9: For the meta-regression related to BRD, diarrhoea and at least one other disorder or treatment.

Fifteen papers evaluating the association between FPT and the above-mentioned outcomes in calves were identified. The main reasons of exclusion were no quantification of risk and no clear definition or quantification of FPT (Fig 1). Most of the papers studied several outcomes, and 68 different models published in the literature were included in the present study. A template for data extraction was drafted that included the numbers of calves and herds studied; the breed (beef or dairy); the statistical method used (logistic regression, Poisson regression, or raw data with contingency table); the expression of risk (relative risk [**RR]** or odd ratio [**OR**]); the prevalence of FPT; the metabolite used to diagnose FPT (total proteins (**TP**) or Ig); the threshold of TP or Ig used to diagnose FTP; the nature (univariate [**U**] or multivariate [**M**]) of the reported model; the prevalence of the outcome or of the mean value if relevant; the duration of the period of outcome inclusion; the value of the risk and its 95% confidence interval (**95% CI**); standard error (**SE**) or standard deviation (**SD**); and all the covariates included within the given model. No distinction was made between total Ig and IgG, and only the designation IgG was used. For diseases, the method of diagnosis (in particular, whether by a veterinarian or a farmer) was also reported in the dataset. Calves were sampled after 24 hour of life, so as to wait the final immunoglobulin absorption. Sampling occurred in a narrow window of time, such as from 24 to 36 hour of age, in particular when TP where measured. When sampling occurred in a large window of time, up to 1 week old, Ig and not TP were sampled in most of case. This was consequently not further considered. All the studied publications had obtained their samples prior to the onset of disease.

The threshold used to diagnose FPT, the presence of a gap between the thresholds used to define calves with or without FPT, the duration of observation of the outcomes, and the diseases analysed in cases of multiple diseases were transformed into categorical moderators for the meta-analysis (FPT_DIAG1_; FPT_DIAG2_; FPT_GAP_; FPT_THRES_; OUT_TIME_ and OUT_MORBIDITY_) according to the rules noted in Table 1. The covariates of the models were not considered further except through the moderators of U/M and OR/RR. These covariates could not be gathered into moderators because of their diversity. Few multivariate models were available. Because of varying degree of dependence amongst the models retained for the meta-regression, 1 extra moderator was created (designated “Group”) for each outcome by grouping the different models in the same paper into different random classes.

A meta-analysis was conducted on the extracted outcomes using the Metafor package of R (version 3.0.2; R Foundation for Statistical Computing, Vienna, Austria). The meta-analysis was performed as previously described [12], and the different steps were recently outlined [13]. Briefly, fixed-effects and random-effects models were first conducted for each outcome to estimate the log-effect size, its 95% CI and its statistical significance. The inconsistency of results among trials was quantified using both Cochran’s Q test and the *I*^2^ statistic [14]. An *I*^2^ value greater than 50% was considered to indicate substantial heterogeneity. If evidence of heterogeneity was found, a meta-regression analysis was subsequently performed to explore the sources of heterogeneity using the log-individual effect size for each trial as the outcome and a fixed-effects or mixed-effects model with the random moderator “Group”. The meta-regression was then conducted by screening for the moderators FTP_DIAG1_, FTP_DIAG2_, FTP_GAP_, FTP_THRES_, OUT_TIME_MORT_, OUT_TIME_RESPI_, OUT_MORBIDITY_, OR/RR, and U/M. The τ^2^ values of the models, with or without moderators, were compared to explain the decrease in heterogeneity that occurred when the moderator was included in the model. All the variables that met the first screening criteria were entered into a backward stepwise regression model until all the variables that remained were significant at P<0.05. Forest plots were used to visually display the estimated effect size, its 95% CI and the final meta-regression adjustments (in grey). For all the meta-regressions, the “Reference” classes of the moderators were chosen to allow for the direct interpretation of the effect size as an adjusted risk of outcome in the case of FPT. Because ORs or RRs were used in the various analyses, the term “risk” may refer to any of these terms. Values within parentheses after the risk values refer to 95% CIs. A sensitivity analysis was finally performed using funnel plots and influential case diagnoses and included the analyses of externally standardized residuals, DFFITS values, Cook's distances, covariance ratios, estimates of τ^2^ and test statistics for (residual) heterogeneity when each study is removed in turn, hat values, and weights for the studies examining the risk of each outcome in the case of FPT.

### Economic model

The economic model was based on the principle of increased risk of disorders (mortality, diseases and ADG decrease) for calves with FPT compared with those without FPT. The methods were previously described for subclinical ketosis [15] and are briefly reported here. The outcome variable of the economic model (*Cost*) was the total cost of FPT for a herd at a given (= study) prevalence of FPT compared with the total cost of FPT for a control herd at a reference prevalence of FPT. The total cost (*Cost*) was estimated by the sum of the cost (*Cost*_*i*_) of each contributor *i* (Eq 1)). Contributors design each of the disorders promoted (mortality and each disease) or performances (ADG losses) modified in the presence of FPT. The calculations were made for an average herd of 100 calves.

$$Cost\;=\;\sum_{i=1}^{1}\;Cost_{i}$$ (1)

For each contributor *i*, *Cost*_*i*_ was the difference between the cost of FPT at the study prevalence (*Cost*_*i*_*FPT*_) and the cost of FPT for the control herd (*Cost*_*i*_*CT*_) (Eq 2).

$$Cost_{i}=Cost_{i\_FPT}-Cost_{i\_CT}$$ (2)

*Cost*_*i*_ was calculated differently for mortality and diseases than for ADG decreases in accordance with the ways the impact of FPT were expressed in the literature. For mortality and diseases, *Cost*_*i*_ was defined as expressed in Eq 3. *Cost*_*i*_ for the study herd was the cost for one calf with disease *i (C*_*i*_ in Eq 3) multiplied by the number of calves suffering from disease *i* denoted by *Unit*_*i*_ (the second part of Eq 3). *Unit*_*i*_ was the difference in the number of calves suffering from disease *i* in the study herd (the first bracket of Eq 3) minus those suffering from the same disease in the control herd (the second bracket of Eq 3). The number of calves suffering from disease *i* in the study herd (within the first bracket) was the number of calves without FPT multiplied by the prevalence of disease *i* within this population (without FPT) plus the number of calves with FPT multiplied by the prevalence of disease *i* within this population (with FPT). The number of calves suffering from disease *i* in the control herd (within the second bracket) was calculated in the same way as for the study herd, but the number of calves with and without FPT was different for the study and control herds.

$$Cost_{i}=C_{i}\times (\left[\left(1-Prev_{HERD\_FPT}\right)\times Prev_{COMPi\_NoFPT}+\;\left(Prev_{HERD\_FPT}\right)\times \;Prev_{COMPi\_FPT}\right]\;-\;\left[\left(1-Prev_{HERD_{CT}}\right)\times Prev_{COMPi\_NoFPT}+\;Prev_{HERD\_CT}\times \;Prev_{COMPi\_FPT}\right])$$ (3)

where

*C*_*i*_ = Unit cost of disease *i* for one animal
*Prev*_*HERD*_*FPT*_ = Prevalence of FPT in the study herd
*Prev*_*HERD*_*CT*_ = Prevalence of FPT in the control herd
*Prev*_*COMPi*_*NoFPT*_ = Within-herd prevalence of disease *i* in calves without FPT
*Prev*_*COMPi*_*FPT*_ = Within-herd prevalence of disease *i* in calves with FPT

*Prev*_*COMPi*_*FPT*_ and *Prev*_*COMPi*_*NoFPT*_ were linked by *RR*_*i*_ (Eq 4).

$$Prev_{COMPi\_FPT}=Prev_{COMPi\_NoFPT}\;\times RR_{i}$$ (4)

where

*RR*_*i*_ = Relative risk of disease *i* in calves with FPT compared to calves without FPT

Combining Eq 3 and Eq 4 led to Eq 5.

$$Cost_{i}=C_{i}\times (\left[\left(1-Prev_{HERD\_FPT}\right)\times Prev_{COMPi\_NoFPT}+\;\left(Prev_{HERD\_FPT}\right)\times \;Prev_{COMPi\_NoFPT}\;\times RR_{i}\right]\;-\;\left[\left(1-Prev_{HERD_{CT}}\right)\times Prev_{COMPi\_NoFPT}+\;Prev_{HERD\_CT}\times Prev_{COMPi\_NoFPT}\;\times RR_{i}\right])$$ (5)

*Prev*_*COMPi*_*NoFPT*_ of Eq 5 was calculated by Eq 6 because often only *Prev*_*COMPi*_*AllPop*_ is available.

$$Prev_{COMPi_{NoFPT}}=100*Prev_{COMPi\_AllPop}\;/\;\left(Prev_{FPT}*\;RRi\;+\;100-Prev_{FPT}\right)$$ (6)

where

*Prev*_*COMPi*_*AllPop*_ = Prevalence of disease *i* in calves with or without FPT
*Prev*_*FPT*_ = Prevalence of FPT from the study reporting *RR*_*i*_ and *Prev*_*COMPi*_*AllPop*_

The cost related to ADG decreased in the case of FPT calculated using Eq 7 for beef, considering the decrease in selling price due to reduced growth.

$$Cost_{ADG\_BEEF}=\Delta_{ADG}\times Duration\;of\;breeding\times Selling\;price$$ (7)

where

*Δ*_*ADG*_ = Decrease in ADG due to FPT (Kg/d)
*Duration of breeding* = Duration between birth and sale for fattening (d.)
*Selling price* = Selling price (€ / Kg of body weight [BW])

For dairy cattle, cost was calculated for heifers considering the extra day before the first insemination in case of decreased ADG, according to Eqs 8 and 9. This allowed us to consider several livestock systems with different values for ADG and age at first calving.

$$Cost_{ADG_{DAIRY}}=\Delta_{DAYS}\times C_{Daily\;Bredding}=\frac{\Delta Weight}{ADG}\times C_{Daily\;Bredding}$$ (8)

$$Cost_{ADG_{DAIRY}}=\frac{\Delta ADG*\left(Age\;in\;d\;at\;calving\;\#1-270\right)}{ADG}\times C_{Daily\;Bredding}$$ (9)

where

*Δ*_*DAYS*_ = Extra days needed to compensate for decreased ADG
*C*_*Daily Breeding*_ = Average daily cost of breeding for a heifer (€ /d)
*Δ*_*Weight*_ = Decrease in weight due to decreased ADG (Kg) for the whole breeding period
*ADG* = Average daily gain for the whole breeding period
*Δ*_*ADG*_ = Decrease in ADG due to FPT

The unit cost of mortality (*C*_*MORTALITY*_) was defined as either the market value of the dairy or beef calf at the date of death (denoted *C*_*MORTALITY*_*Replacement*_) or the lost earnings (denoted $C_{{MORTALITY}_{Earningforgone}}$) up to the date of sale for beef calves (Eq 10).

$$C_{MORTALITY_{Earningforgone}}=Selling\;Price\times Selling\;Weight-C_{Conc}\;*\;Qty_{Conc}$$ (10)

where

*Selling Weight* = Weight of the calf at sale (Kg)
*C*_*Conc*_ = Cost of concentrate for calves (€ /Kg)
*Qty*_*Conc*_ = Quantity of concentrate eaten per calf for the whole breeding period (Kg)

The unit cost of morbidity (*C*_*MORBDITY*_) was the sum of 3 components proportional to weight at treatment (i.e., drugs), fixed per animal (i.e., veterinarian visits) and labour of the farmer (Eq 11).

$$C_{MORBIDITY}=C_{MORBIDITY\_Prop}+\;C_{MORBIDITY\_Fixed}\;+C_{MORBIDITY\_Labour}$$ (11)

where

*C*_*MORBIDITY*_*Prop*_ = Proportional cost of one case of morbidity (€ / Kg BW)
*C*_*MORBIDITY*_*Fixed*_ = Fixed cost of one case of morbidity (€ /case)
*C*_*MORBIDITY*_*Labour*_ = Labour cost of one case of morbidity (€ /case)

In cases of diarrhoea and BRD, the risk of relapse has to be accounted for. *Unit*_i,_ calculated in Eq 5, only considers calves that are sick (and treated) at least once. Therefore, the relapse risk was taken in account through *C*_*MORBIDITY*_, according to Eq 12 and Eq 13. *C*_*DIARRHOEA*_*relapse*_ and *C*_*BRD*_*relapse*_ were included in Eq 5 instead of *C*_*DIARRHOEA*_ and *C*_*BRD*_, respectively.

$$C_{DIARRHOEA\_relapse}=C_{DIARRHOEA}\;(1+{Relapse}_{1_{DIARRHOEA}})$$ (12)

$$C_{BRD\_relapse}=C_{BRD}\;(1+{Relapse}_{1_{BRD}}\;)\;(1+\;{Relapse}_{2_{BRD}}\;)$$ (13)

where

${Relapse}_{1_{DIARRHOEA}}$ = Relapse risk of rank 1 for diarrhoea
${Relapse}_{1_{BRD}}$ = Relapse risk of rank 1 for BRD
${Relapse}_{2_{BRD}}$ = Relapse risk of rank 2 for BRD

To account for more severe disorders for calves with FPT compared to these without FPT and for supplementary difficulties in managing the herd in cases of high *Prev*_*HERD*_*FPT*_, 2 conditional coefficients of severity (Eqs 14 and 15) were introduced in Eq 5.

$$C_{MORBIDITY}\;=\{\begin{array}{c} C_{MORBIDITY}\;*\;Sev_{FPT}\;for\;calves\;with\;FPT\\ C_{MORBIDITY}\;for\;calves\;without\;FPT \end{array}$$ (14)

$$C_{MORBIDITY}\;=\{\begin{array}{c} C_{MORBIDITY}\;*\;Sev_{PRESSURE}\;when\;Prev_{HERD\_FPT}\;\geq 40\%\\ C_{MORBIDITY}\;when\;Prev_{HERD\_FPT}<40\% \end{array}$$ (15)

Eqs 12, 13, 14 and 15 apply additively in Eq 5.

The economic model was run using Scilab open source software (www.scilab.org) with 10,000 iterations, and 95% prediction intervals (PI) and 95% CIs were calculated. The PI makes it possible to predict the situation for the next farm with 95% probability, whereas the CI indicates the situation in 95 of 100 farms visited.

The *RR*_*i*_, the prevalence and the unit costs *C*_*i*_ used in the assessment are presented in Table 2 and explained in detail. Most of the input parameters were included as a law of distribution (normal or log-normal) and not as a point estimate. Four scenarios were proposed that combined different input parameters. The baseline scenario was the most probable. The alternative scenario included more contributors and different calibrations. The low and high scenarios used minimum and maximum values for calibration, respectively. For *RR*_*i*_, the alternative and high scenarios included raw data from the literature review before any correction, whereas the baseline and low scenarios included the results of the above meta-analysis. *Prev*_*COMPi*_*NoFPT*_ was calculated using Eq 6 and applied to studies of the meta-analysis (S1–S3 Tables). Lack of data on omphalitis and septicaemia led to a definition in line with the entity “morbidity” of the meta-analysis. *Prev*_*Mortality*_*NoFPT*_ was defined based on Eq 6, and data from studies included in the meta-analysis led to a value considered low by the authors (3.2%). *Prev*_*Mortality*_*AllPop*_ is well described for France. On average, it is 6.2% and 10% between birth and 1 month of age for beef and dairy cattle, respectively (Raboisson, 2014). *Prev*_*Mortality*_*NoFPT*_ is shown in Table 2 with the 2 last values included in Eq 6, and *Prev*_*FPT*_ was fixed at 25% and 40% for beef and dairy cattle, respectively.

**Table 2:** Input parameters for the economic model.

		Scenario			
	Law^1^	Baseline	Alternative	Low	High
*RR*_*i*_ or impact of FPT					
Mortality	LN	2.12 (0.19)	2.41 (0.20)	2.12 (0.19)	2.41 (0.20)
BRD ^2^	LN	1.75 (0.08)	2.27 (0.17)	1.75 (0.08)	2.27 (0.17)
Diarrhoea	LN	1.51 (0.18)	1.81 (0.07)	1.51 (0.18)	1.81 (0.07)
Omphalitis	LN	///	1.91 (0.08)	///	1.91 (0.08)
Septicaemia	LN	///	1.91 (0.08)	///	1.91 (0.08)
*∆ADG* ^2^^**,**^^3^ (g/d)	N	54 (48)	81 (76)	54 (48)	81 (76)
Prevalence in populations without FPT					
$Prev_{MORTALITY_{NoFPT}}$ (%)	//	0.048 ^4^/ 0.069 ^5^			
$Prev_{BRDY_{NoFPT}}$^3^ (%)	N	0.283 (0.127)			
$Prev_{DIARRHOEA_{NoFPT}}$^3^ (%)	N	0.227 (0.127)			
$Prev_{OMPHALITIS_{NoFPT}}$ (%)	//	0.05			
$Prev_{SEPTICAEMIA_{NoFPT}}$ (%)	//	0.03			
Unit cost (*C*_*i*_)					
*C*_*MORTALITY Remplacement*_ ^3^ (dairy, €)		125 (9)	125 (9)	45 (5)	330 (15)
*C*_*MORTALITY Remplacement*_ ^3^ (beef, €)		375 (22)	///	375 (22)	///
$C_{MORTALITY_{Earningforgone}}$^6^ C_conc_ (€/ton)	///	///	250/125/175	///	250/125/175
$C_{MORTALITY_{Earningforgone}}$^6^ Selling Price^3^ (€/Kg BW)	N	///	2.4 (0.13)/3.0 (0.12)/ 2.56 (0.038)	///	2.4 (0.13)/3.0 (0.12)/2.56 (0.038)
$C_{MORTALITY_{Earningforgone}}$^6^^,^ Selling Weight (Kg BW)	///	///	337/285/374	///	337/285/374
$C_{MORTALITY_{Earningforgone}}$^6^ Qty_Conc_ (Kg)	///	///	290/141/400	///	290/141/400
C_BRD_Prop_^3^ (€/100 Kg BW)	N	17.6 (3.8)	17.6 (3.8)	10.31	25.31
C_Diarrhoea_Prop_^3^ (€/100 Kg BW)	N	17.6 (4.1)	17.6 (4.1)	10.45	26.65
C_Diarrhoea_Fixed_^3^ (€)	N	40.0 (7.6)	40.0 (7.6)	30	60
C_Omphalitis_Prop_^3^ (€/100 Kg BW)	N	8.5 (1.5)	8.5 (1.5)	5	11
C_Omphalitis_Fixed_^3^ (€)	N	150 (25.5)	150 (25.5)	100	200
C_Septicaemia_Prop_^3^ (€/100 Kg BW)	N	27.84 (3.8)	27.84 (3.8)	19.2	40.2
C_*Daily Breeding*_ (€/day)	///	0.74	1.28	0.74	1.28
*Selling Price*, *Beef* ^3^ (€/Kg BW)	N	2.56 (0.04)	2.70 (0.15)	3.00 (0.12)	2.40 (0.13)
Technical parameters					
Body weight of dairy calves ^3^^**,**^^7^ (Kg)	N	57.5 (8.9)	57.5 (8.9)	40	75
Body weight of beef calves ^3^^**,**^^7^ (Kg)	N	75.0 (12.7)	75 (12.7)	50	100
ADG, dairy ^3^ (g)		615 (18)	615 (18)	615 (18)	615 (18)
Selling age, beef (months)	///	8.5	8.3	6.5	10.0

1: LN = LogNormal, N = Normal

2: Average daily gain reduction due to FTP and bovine respiratory diseases

3: mean (and SD)

4: Beef

5: Dairy

6: formula is selling price * selling weight–C_Conc_ * Qty_Conc_; only applies to beef cattle

7: particularly during treatment.

Unit costs (*C*_*i*_) were derived from different sources. These have been explained in detail elsewhere [15–17]. *Selling Price*, *Selling Weight*, *Qty*_*Conc*_, *Age in d at calving* #1, *C*_*Daily Bredding*_ and *Duration of breeding* were adapted from French Livestock Institute publications [18,19]. *C*_*MORTALITY Remplacement*_ was the market value [20]. *C*_*MORBIDITY*_ was defined by the authors based on health consequences and common medical protocols. Based on the authors’ expertise, *C*_*i*_ is explained in S1 Table. Medicines were chosen according to the most common protocols observed in the field, and prices were defined as the sale prices recommended by veterinary clinics in 2014 (www.centravet.fr).

*Prev*_*HERD*_*CT*_ was defined at 10%. *Sev*_*FPT*_ and *Sev*_*PRESSURE*_ were fixed at 1.375, according to one or two extra days of treatment (mean = 4 days) needed for calves with FPT compared to these without [21]. ${Relapse}_{2_{DIARRHOEA}},\;{Relapse}_{1_{BRD}}$ and ${Relapse}_{1_{BRD}}$ were 20%, 30% and 20%, respectively.

## Results

### Meta-regression

**Fig 2:**
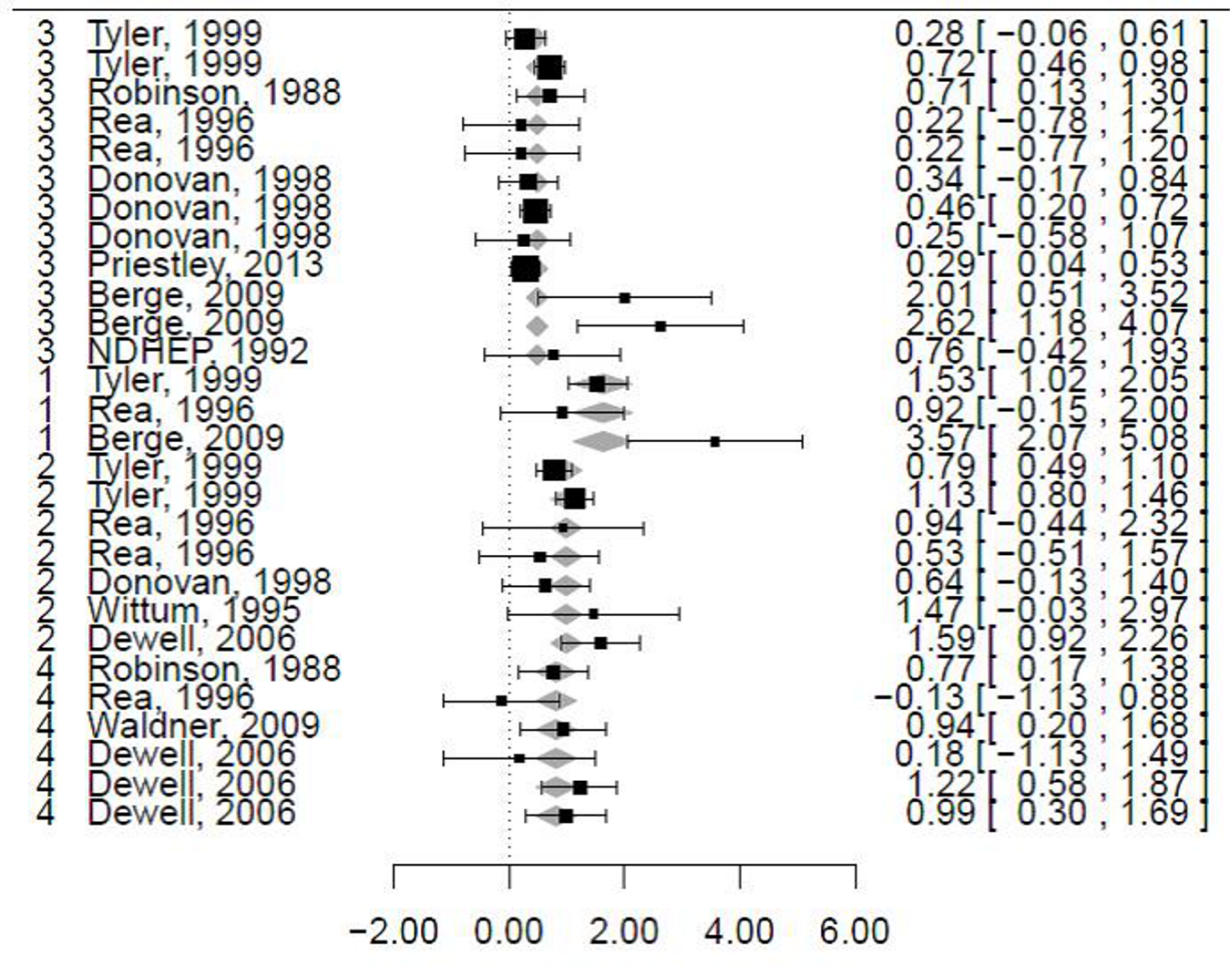
Forest graph for mortality. Adjustments were made for the moderator FPT_DIAG1_. The column on the left refers to the values of FPT_DIAG1_. The column on the right refers to the log-scale observed outcomes (OR or RR) and their relative 95% CIs. The grey squares represent the log-effect size adjusted for FPT_DIAG1_.

The association between FPT and mortality was reported in 28 models from 10 publications (S2 Table). The mean (SD) risk of mortality associated with FPT was 4.87 (7.62). The heterogeneity of the dataset was high (I^2^ = 68% [95% CI = 46–79] and Q statistics χ^2^ = 80, df = 27, *P* < 0.001). The intercept of the log-effect size in the mixed-effects model with no moderator was 0.88 (SE = 0.020, P<0.001), which corresponded to an effect size of 2.41 (1.62–3.58). Including the moderators FTP_DIAG1_, FTP_DIAG2_ and FTP_GAP_ reduced the heterogeneity by 23, 29 and 22%, respectively (Table 3). All other moderators were not significant (P>0.05), and their inclusion in the meta-regression did not decrease the heterogeneity. Similarly, no multivariate meta-regression allowed us to reduce the heterogeneity. Replacing FTP_DIAG1_ with FTP_DIAG2_ in the meta-regression led to an OR of mortality in the case of FPT of 1.92 (data not shown) instead of 2.12 (Table 3). The sensitivity analysis showed no outlier for the meta-regression without a moderator, but doubt exists regarding model #4 (S2 Table) when the moderator FTP_DIAG2_ was considered based on DIFFTS and cov and τ^2^ statistics. Excluding these data did not lead to significant changes (Table 3). In summary, the present work retains the risk (95% CI) of mortality in calves with FPT adjusted for the moderator FTP_DIAG2_ (Fig 2), which is 2.16 (1.51–3.09).

**Table 3:** Risk of outcomes in cases of FPT obtained with the meta-analysis.

	Descriptive statistics^1^			Mixed-effects meta-regression^1^				
	n	m	SD	Estimate (SE) and P value			Risk	[95% CI]
MORTALITY								
No moderator	28	4.08	6.69	0.88	(0.20)	***	2.41	[1.62–3.58]
Intercept				0.77	(0.18)	***	2.16	[1.51–3.09]
FPT_GAP_ = 0	18	2.60	2.80	Reference				
FPT_GAP_ = 1	6	9.30	14.20	0.50	(0.12)	***	1.64	[1.30–2.08]
Intercept				0.75	(0.19)	***	2.12	[1.43–3.13]
FPT_DIAG1_ = 1	3	14.24	18.40	1.01	(0.24)	***	2.74	[1.71–4.39]
FPT_DIAG1_ = 2	7	3.10	1.47	0.40	(0.13)	**	1.49	[1.15–1.92]
FPT_DIAG1_ = 3	12	3.07	3.79	Reference				
FPT_DIAG1_ = 4	6	2.15	0.95	-0.04	(0.24)		0.96	[0.60–1.53]
Intercept^2^				0.74	(0.20)	***	2.10	[1.43–3.13]
FPT_DIAG1_ = 1^2^				1.11	(0.49)	***	3.03	[1.16–7.92]
FPT_DIAG1_ = 2^2^				0.40	(0.13)	**	1.49	[1.15–1.92]
FPT_DIAG1_ = 3^2^				Reference				
FPT_DIAG1_ = 4^2^				-0.04	(0.24)		0.96	[0.60–1.53]
BRD (1)								
No moderator	12	2.24	1.20	0.82	(0.17)	***	2.27	[1.62–3.17]
No moderator^2^	11			0.55	(0.08)	***	1.75	[1.50–2.03]
DIARRHOEA (2)								
No moderator	5	1.70	0.70	0.56	(0.31)	.	1.76	[0.94–3.27]
No moderator^2^	4	1.85	0.30	0.41	(0.18)	***	1.51	[1.05–2.17]
AT LEAST ONE DISORDER OR TREATMENT (3)								
No moderator	8	2.72	1.71	0.58	(0.17)	***	1.80	[1.28–2.52]
MORBIDITY (1+2+3)								
No moderator	25	2.34	1.31	0.64	(0.08)	***	1.91	[1.63–2.24]
Intercept				0.79	(0.13)	***	2.21	[1.74–2.81]
FPT_DIAG1_ = 1	5	2.24	0.83	-0.07	(0.14)		0.93	[0.70–1.22]
FPT_DIAG1_ = 2	6	2.94	1.80	0.01	(0.19)		0.10	[0.69–1.46]
FPT_DIAG1_ = 3	8	2.27	1.46	Reference				
FPT_DIAG1_ = 4	6	1.92	0.87	-0.44	(0.15)	**	0.64	[0.47–0.86]
Intercept				0.69	(0.22)	**	1.99	[1.28–3.10]
OUT_MORBIDITY_ = 1	8	2.72	1.70	Reference				
OUT_MORBIDITY_ = 2	5	1.71	0.70	-0.73	(0.28)	*	0.48	[0.28–0.83]
OUT_MORBIDITY_ = 3	12	2.36	1.19	0.21	(0.26)		1.23	[0.74–2.05]

1: n: number; m: mean; SD: standard deviation; SE: standard error; 95% CI: 95% confidence interval. P values are coded as follow

***P<0.001

**P<0.01

*P<0.05

.P<0.10.

2: Meta-regression after the sensitivity analysis and exclusion of one model of the data.

**Fig 3:**
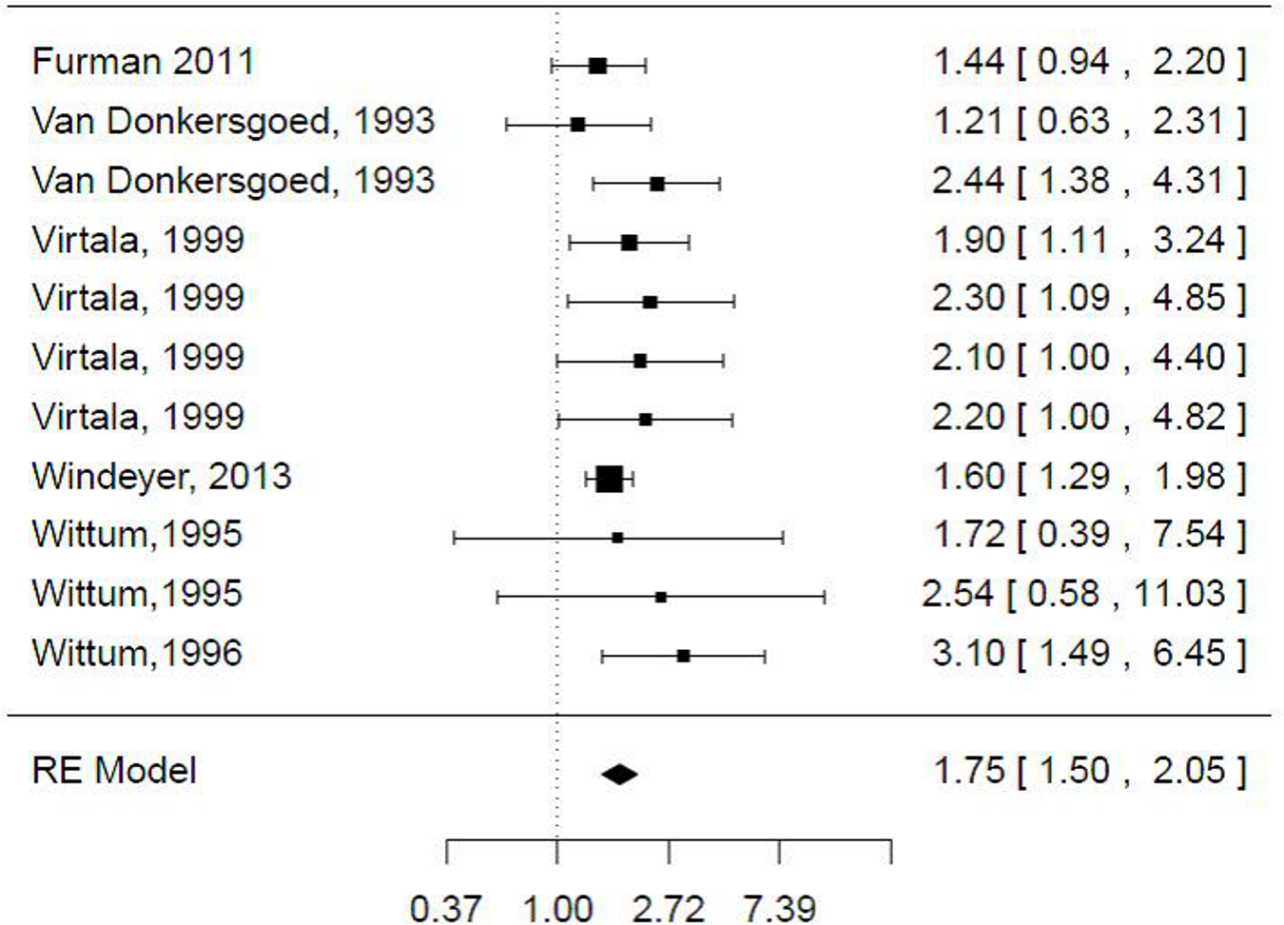
Forest graph for bovine respiratory disease. The column on the right refers to the observed outcomes (OR or RR) and their relative 95% CIs.

The association between FPT and BRD was reported in 12 models from 5 publications (S3 Table). The mean (SD) risk of BRD associated with FPT was 2.24 (1.20). The heterogeneity of the dataset was high (I^2^ = 72% [55–82] and Q statistics χ^2^ = 61, df = 11, *P* < 0.001). The intercept of the log-effect size in the mixed-effects model with no moderator was 0.82 (SE = 0.17, P<0.001), which corresponded to an effect size of 2.27 (1.62–3.17). No moderators were significant or allowed a decrease in heterogeneity. The sensitivity analysis showed an outlier (model #2) based on most of the statistics considered. Excluding these data led to a decrease in the risk (95% CI) of BRD in calves with FPT (Fig 3 and S1 Fig), which was 1.75 (1.50–2.03). I^2^ also decreased.

The association between FPT and diarrhoea was reported in 5 models from 2 publications (S1 Table). The mean (SD) risk of BRD associated with FPT was 1.71 (0.70). The heterogeneity of the dataset was high (I^2^ = 80%). The intercept of the log-effect size in the mixed-effects model with no moderator was 0.56 (SE = 0.32, P = 0.07), which corresponded to an effect size of 1.76 (0.94–3.27). None of the moderators were significant. Excluding the data from model #2 as suggested by the sensitivity analysis led to a risk (95% CI) of diarrhoea in calves with FPT of 1.51 (1.05–2.17).

Four publications (8 models) provided data on the risk of at least one disorder (except mortality) or at least one treatment in cases of FPT (S3 Table). The mean (SD) risk associated with FPT was 2.72 (1.71). I^2^ was 51%. The intercept of the log-effect size in the mixed-effects model with no moderator was 0.58 (SE = 0.17, P<0.05), which corresponded to an effect size of 1.80 (1.28–2.52). None of the moderators were significant. The sensitivity analysis did not reveal any outliers.

When combining data related to the last 3 outcomes, 25 studies from 10 publications were available (Table 3). The mean (SD) risk of BRD associated with FPT was 2.34 (1.31). The heterogeneity of the dataset was high (I^2^ = 73% [50–88] and Q statistics χ^2^ = 107, df = 24, *P* < 0.001). The intercept of the log-effect size in the mixed-effects model with no moderator was 0.64 (SE = 0.08, P<0.001), which corresponded to an effect size of 1.91 (1.63–2.24). The moderators FTP_DIAG1_ and OUT_MORBIDITY_ were significant, but their inclusion did not reduce the heterogeneity and led to slight changes in the log-effect size (Table 2).

The ADG change related to FPT was reported in 12 models from 6 publications (S4 Table). The mean (SD) ADG decrease for calves with FPT was 81 g/day (76). It was 54 (48) when redundant data from Robinson, 1998, were excluded (only the ADG for the period of 0 to 180 days was considered). SEs of the models were not provided in most of the results, preventing a meta-regression for this outcome. The ADG change relative to morbidity was reported in 6 models from 4 publications. No further analysis was provided.

### Economic assessment

**Fig 4:**
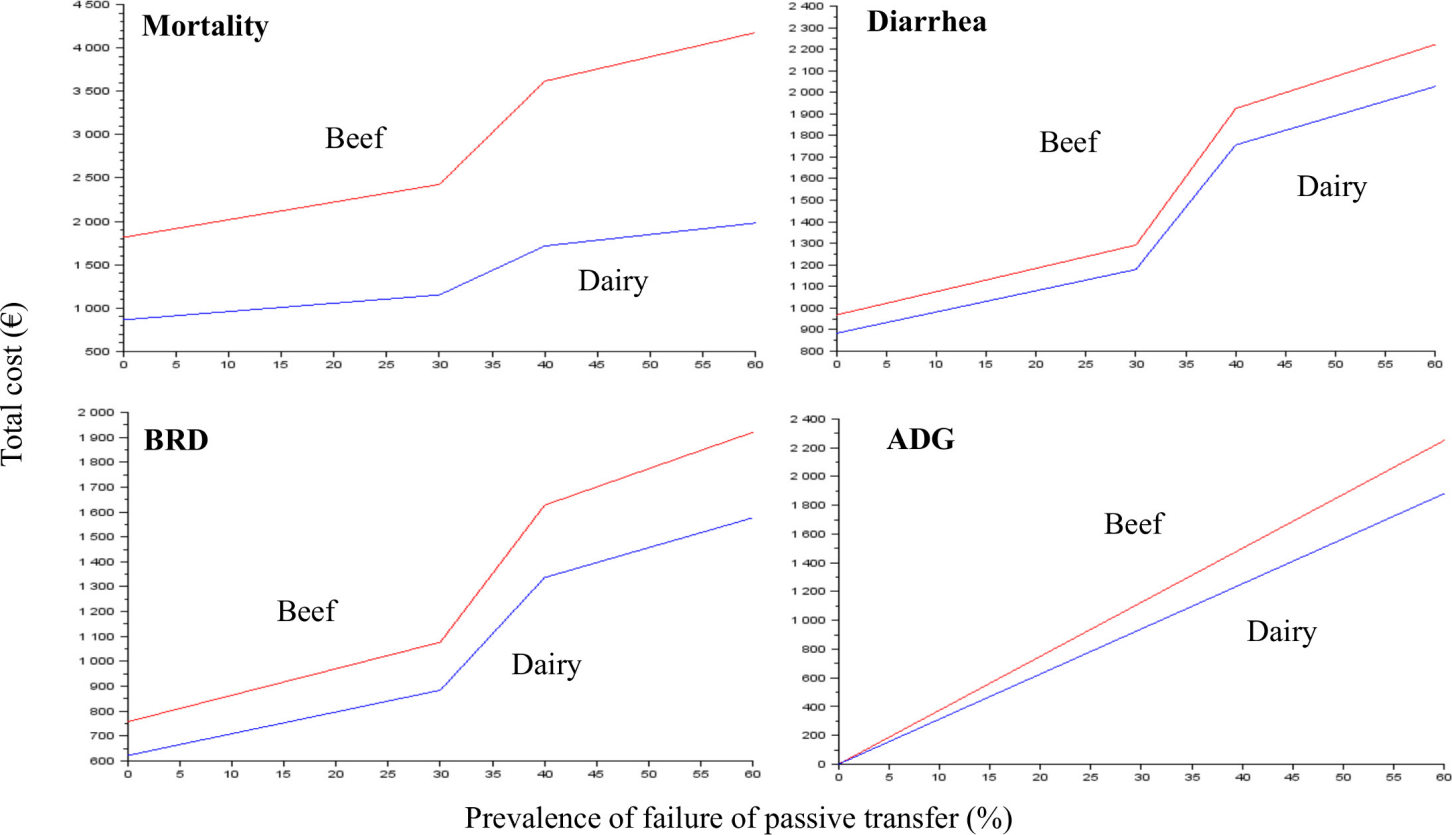
The total cost of the contributors in cases of FPT for beef and dairy in the baseline scenario. BRD: bovine respiratory disease; ADG; average daily gain.

The total cost of one case of FPT in the baseline scenario is €60 [95% PI = €10–109] and €80 [95% PI = €20–139] per dairy and beef calf, respectively. It is €95 [95% PI = €38–151] and €132 [95% PI = €70–200] in cases of high prevalence (Table 4). These costs are nearly twice as high in the alternative scenario, up to three times as high in the high scenario, and approximately 10–20% lower in the low scenario. The total cost of each contributor increases linearly up to a prevalence of 40%, and above 40% the slopes differ in accordance with the *Sev*_*PRESSURE*_ applied. One exception is ADG (Fig 4). The relative parts of each contributor (Table 5) for the different scenarios show the high part related to ADG compared to other parts. This is most commonly observed in the alternative and high scenarios in accordance with the higher ∆ADG (Table 3), and for dairy compared to beef cattle. This part of the ADG tends to decrease with a high prevalence of FPT. The total cost of FPT increased by 20% and 40% for dairy heifers that first calved at 2.5 or 3 years old, respectively, compared to those that calved at 2 years old (results not shown); the difference originated from the component ADG, which was applied to the whole breeding period.

**Table 4:** Total cost of FPT under several scenarios^1^.

	Dairy			Beef		
	Mean costs (€) per calf with FPT	Variation	95% PI (€)	Mean costs (€) per calf with FPT	Variation	95% PI (€)
For low prevalence of FPT								
Baseline	60		10–109	80		20–139
Alternative	121	+100%	1–246	140	+72%	43–233
High	52	-13%	4–101	72	-10%	19–125
Low	180	+300%	46–319	187	+233%	77–296
For high prevalence of FPT								
Baseline	95		38–151	132		70–200
Alternative	162	+70%	32–292	225	+170%	120–330
High	78	-18%	22–133	121	-9%	60–181
Low	285	+300%	133–438	323	+244%	183–432

1: The baseline scenario was the most probable. The alternative scenario was also possible and included higher prices for some contributors. The high and low scenarios minimized and maximized all input parameters.

**Table 5:** Some contributors to the total cost.

		Dairy			Beef			
	Mortality	BRD	Diarrhoea	ADG	Mortality	BRD	Diarrhoea	ADG
For low prevalence of FPT (mean and [95% PI])								
Baseline	€9.6	€9.6	€8.8	€33.1	€20.5	€10.7	€10.7	€37.7
	[7–12](	[0–19]	[0–25]	[29–58]	[16–24]	[0–23]	[0–28]	[25–50]
	(15%)	(13%)	(17%)	(55%)	(26%)	(16%)	(13%)	(45%)
Alternative^1^	€11.2	€12.6	€13.	€83.8	€47.8	€15.4	€14.3	€58.7
	[8–14]	[2–23]	[0–29]	[55–112]	[40–55]	[2–28]	[0–32]	[38–78]
	(8%)	(11%)	(10%)	(68%)	(30%)	(10%)	(10%)	(41%)
For high prevalence of FPT (mean and [95% PI])								
Baseline	€22.3	€18.7	€22.6	€33.1	€47.0	€22.9	€24.8	€37.7
	[15–30]	[0–53]	[0–75]	[0–126]	[30–63]	[0–71]	[0–89]	[0–110]
	(23%)	(12%)	(22%)	(35%)	(34%)	(17%)	(17%)	(27%)
Alternative^1^	€24.0	€24.3	€27.3	€83.8	€101.0	€29.9	€29.7	€58.0
	[19–28]	[0–60]	[0–78]	[0–420]	[68–135]	[0–83]	[0–101]	[0–172]
	(14%)	(12%)	(14%)	(51%)	(37%)	(12%)	(11%)	(26%)

1: The sum is not 100% because the contributors omphalitis and diarrhoea are not shown.

## Discussion

### Meta-regression

The present work used the OR or RR. Differences between OR and RR are negligible when the probability of the outcome is low and when the baseline risk for each subgroup is relatively constant [22]. These criteria were met for mortality in the present work but remained questionable in terms of morbidity. A scarcity of data led us to consider all available data. Regressions including the moderator RR/OR were not significant.

The definition of FPT was based on low TP or low IgG. At present, TP and IgG are commonly and consensually used to evaluate FPT. Both are quantitative indicators of immune transfer success, although TP may increase due to inflammation and rather than to IgG pinocytosis. The thresholds for TP and IgG used to define FPT vary, but thresholds used as references in the present work are those most commonly used. Subclinical disorders associated with diseases or production changes are defined with a threshold of metabolites or of biochemical parameters that maximize the sensitivity and specificity of the diagnosis. These studies simultaneously defined the objective animal-level threshold of interest parameters and the risk linked to the disease or production change. Similar intensive studies have been conducted for subclinical ketosis but rarely for FPT. Except for a few examples [23] that only involved small populations, no multiple regression with different thresholds and associated ROC curves has been performed for FPT. The threshold to define FPT is consequently often arbitrary and without justification. This highlights the need to standardize the risk value by the definition of FPT, as we have done here. This is in accordance with the results of the meta-regression for mortality and morbidity showing the significance of the moderator FTP_DIAG_ and the decrease in heterogeneity when this moderator was applied (Table 3). The adjustment of the mean risk by the threshold used to diagnose FPT allowed us to prevent an overestimated mean risk (2.12 instead of 2.41) in the present work (Table 2). Similarly, the risk of mortality for FPT comparing two extreme populations for IgG or PT is overestimated (FPT_GAP_, Table 2). The duration of observation of mortality was not reported as a significant bias of the risk value in the present work, even though the longer the study, the higher the expected mortality.

The absence of any significant moderator for BRD, diarrhoea, or at least one other disorder is linked to the low statistical power permitted by the size of the dataset. The risk of diarrhoea in cases of FPT is extremely low. This is in accordance with the high prevalence of diarrhoea in calves without FPT, which mathematically caps the value of the risk. The risk for BRD in cases of FPT is of the same magnitude as the risk of diarrhoea. Improvement of BRD through colostrum feeding is rarely promoted in the field as opposed to diarrhoea. A recent study has shown that vaccinating cows against BRD before calving and good colostrum feeding help to control BRD in calves [24].

One other significant limitation of the present meta-regression originates from the data available, particularly (i) the lack of co-variates in many of the raw models included, and (ii) the lack of data for the other consequences of FPT that were not included here. First, the value of the risk obtained in the various studies depends on not only the thresholds used but also the adjustments made by potential biases of co-factors. For example, no model explaining the risk of diarrhoea in cases of FPT included the presence of BRD (or versa); instead, the interaction between both is described [25]. The OR of mortality in cases of FPT was adjusted by neither diarrhoea nor BRD in the studies, preventing any adjustment within the meta-regression. Second, the disorders and production changes associated with FPT in only a few studies were not included in the present work. This includes feed efficiency, age at first calving, milk production at first and second lactation, and risk of culling at first lactation [26–28]. Some of these factors are related to ADG. Data on the reduction of ADG in cases of FPT is controversial, with significant differences between results. Interestingly, most of the high values originate from one publication [29]. Only 3 studies reported ADG calculated using a long duration, but ADG variability still remains (16, 50 and 134 g/d). Although most of the models involved are multivariate, they did not include morbidity as a co-variate. This may bias the results and contribute to the significant heterogeneity of the estimations.

The present meta-regression was performed as previously described [12,30]. The intercept obtained in the random-effects model with no moderator was more precise than the raw mean of risk because the lower the variance of the raw value, the higher the weight in the meta-regression. These changes may be high. For example, the OR for mortality decreased from 4.87 (raw mean) to 2.41 (mixed-effects meta-regression without moderator, Table 3). The final meta-regression (and relative adjusted risk) that was retained was judged on the reduction of the heterogeneity relative to the regression without moderators. Only the mixed-effects models were reported in the present work, in accordance with the structure of the dataset (several lines of the dataset were from the same publication). The funnel plots did not show any publication bias. They were not asymmetric, suggesting no studies with a reduced likelihood of inclusion within the meta-analysis because they were not published due to small or non-significant results.

### A double-step stochastic method for easy application of the results in the field

In the cattle industry, many decisions about animal health are made without considering economics. In particular, estimates of the cost of multiple diseases thought to be occurring simultaneously -where losses or extra costs are difficult to attribute to one disease or the other- are rare. The proposed double-step approach has been extensively discussed elsewhere [15]. It allowed the costs of diseases with multiple interactions to be estimated accurately because of (i) the correction permitted by the meta-analysis that led to a reduced risk of economic miscalculation due to overestimated or underestimated RR and (ii) the use of the prevalence of diseases in calves without FPT instead of the prevalence in calves with and without FPT. These two types of adjustments to the input parameters allowed for the proposed accurate estimation. The total cost of FPT was considered to be the sum of the costs related to mortality, morbidity and ADG decrease. Such an approach is accurate because the input parameters are adjusted by the other components. For example, mortality represents the whole mortality linked to FPT regardless of the cause of the mortality, and the cost of morbidity never includes consecutive deaths from diarrhoea, BRD or other causes. This is why the unit cost of morbidity was based on extra costs only and did not include losses in production. Another example is ADG, which included not only the potential direct impact of FPT but also the indirect impact through morbidity. The choices were made according to the data that were available and have no connection to biological mechanisms. The way the economic model was constructed has to be kept in mind when interpreting the weight of the different components in the total cost of FPT. Because FPT appears around birth and generally has short-term consequences, the static approach proposed seems adequate. A dynamic model may have been useful but it would have led to significant difficulties in calibration.

The present results were expressed as the total cost of one case of FPT, but the avoidable cost linked to FPT can easily be estimated in the field. Our detailed method is explained elsewhere [15]. Briefly, the prevalence of FPT at the initial stage must be estimated first (using TP, for example). The total avoidable cost for a given farm will then be (i) the unit cost of FPT multiplied by (ii) the number of calves born (iii) multiplied by the prevalence of FPT at the initial stage minus a reference (not null) prevalence of FPT. Whether the expected prevalence (based on practitioner experience) after measures have been adopted is used instead of the reference prevalence, the comparison of the avoidable cost linked to FPT and the prices of the measures proposed helps in making a decision. Using the stochastic approach, both the mean and a 95% PI have been proposed. Because of biological variability, the use of the mean values as well as the ranges of the 95% PI is recommended to make robust economic-oriented decisions when end users calculate the herd-level avoidable costs. Farm advisors must use the PI rather than the CI because the PI predicts the value with 95% probability for the next case (i.e.; the next herd they have to evaluate), whereas the CI gives the distribution of values for 95 of the next 100 cases.

### Calibration and interpretation of the economic model

Despite the significant efforts made in the present study to address double counting and incorrect estimations, factors such as ADG, septicaemia and omphalitis continued to be difficult to address. Some assumptions were also adopted when performing the current estimation due to a lack of data. A good understanding of the calibration of the economic model and the report on the part of each contributor (Table 5) allow an integrative use of the present results.

First, the model was performed at the herd level to allow for different calibrations of low and high prevalences of diseases. The importance of the infectious pressure on the management and control of disease at the herd level cannot be overlooked. The values of *Sev*_*PRESSURE*_ and *Prev*_*HERD*_*FPT*_ that may affect the infectious pressure (Eq 15) remain questionable. The present method may present results at the herd level (mean cost per 100 cows for a given prevalence) or per cow with FPT, as is the case here. The total cost and marginal cost were the same up to an FPT prevalence of 40%. This result is consistent with how the total cost was calculated based on the input parameters and the literature. Above this prevalence, the marginal cost of FPT increased dramatically. The threshold established at an FPT prevalence of 40% definitely does not exist in the field, and infectious pressure may be progressive. It is the responsibility of the user of the present results in the field to decide which results to use (i.e., 60 or 120 for dairy and 80 or 160 for beef) according to the farm and the current situation.

Second, the clear identification of the relevant part of each contributor allows for the modulation of the results to various farming systems. Because the different contributors to the total cost of FTP are additive, the total cost can easily be recalculated to best fit to the user’s experience and the farming system. The summary of the input parameters (Table 3) and parts of the contributors for all key situations (Table 5) can assist in such work. For example, the contributor ADG may be adapted depending on the user’s goal. ΔADG applies for the whole breeding period for both dairy and beef calves. For beef calves, ΔADG is likely to apply up to weaning, and beef calves are mostly sold at this point. Reduced ADG after weaning and selling in case of FPT was not considered even though it may occur. In other words, the present total cost is appropriate for breeding activity only and excludes fattening activity. Similarly, ΔADG was not applied to male dairy calves, which are fattened outside the farm of birth in most cases. By contrast, the application of ΔADG for the whole breeding period for dairy heifers may be excessive, and it might only apply for 6 months to one year. This suggests that the cost for calving at 2.5 or 3 years is the same as for a first calving at 2 years. The literature suggests that weight curves of heifers with FPT are lower than those without FPT, but the curves remain parallel [28]. This would indicate that only-half or one-quarter of the contributor ADG needs to be considered for dairy cattle. Other possibilities for the integrative use of the present results are the differentiation of male and female cattle on a dairy farm: deleting the ADG contributor for males and just considering it for females is a possible way to apply the present total cost. Such an approach may be appropriate for dairy farms with differentiated habits in colostrum distribution (and care provided) for males and females. By contrast because of the lack of consistency on the value of ΔADG, using the alternative or high scenario instead of the baseline or low scenario is not recommended for the contributor ADG. The total costs for dairy males and females with FPT are consequently €28.2 [95% PI = 9.1–47.2] and €51.1 [95% PI = 29.4–72.9], respectively, when applying the present parameters for ADG (only ADG in females, and reduced to one-quarter).

By contrast, questions have arisen on a potential underestimation of diarrhoea and BRD contributors in the baseline and low scenarios. On one hand, the unit cost of these contributors is quite low when compared to the real cost, including the intervention of a veterinarian. On the other hand, only some ill calves are observed by practitioners in most farming systems, and the definition of the unit cost accounted for that (S1 Table). The inclusion of the contributors septicaemia and omphalitis should remain at each practitioner’s discretion. A different approach may be used in the field for these 2 contributors: septicaemia may be related to high mortality, and omphalitis is often well identified, allowing for a specific evaluation of the treatment cost.

Third, some key parameters are poorly known. Relapse after BRD or diarrhoea and the value of Sev_PRESSURE_ may differ greatly among farming systems, farms, dominant pathogens involved and years.

## Conclusions

The present work proposed an adjusted risk of mortality, diarrhoea, and BRD in cases of FPT. These results may be used by practitioners and also for the further calibration of epidemiologic or economic empiric models. The mean total costs related to FPT for farmers were estimated to be €60 and €80 per dairy and beef calf with FPT, respectively. The 95% PIs were €10–109 and €20–139, respectively. As a result of using a double-step stochastic method, the proposed economic estimation constitutes a first estimate available for FPT. Nonetheless, a lack of data for calibration limits confirmation of the accuracy of the present estimation. The way in which the results are presented facilitates their use in the field and allows, with limited effort, the combination of the cost of each contributor to increase the appropriateness of the economic assessment to the situations farm-advisors may face. The present economic estimates are also an important tool to evaluate the profitability of measures that aim at improving colostrum intake.

## Supporting Information

S1 Fig Example of an influential case diagnostics graph for respiratory diseases (PDF)

S1 Table Definitions of costs of diseases (PDF)

S2 Table Raw data used for the meta-analysis and to calculate the prevalence of mortality in calves without failure of passive transfer (PDF)

S3 Table Raw data used for the meta-analysis and to calculate the prevalence of diseases in calves without failure of passive transfer (PDF)

S4 Table Raw data used for the review of ADG reduction in calves without failure of passive transfer (PDF)
