# Supplementary material for: Failure of Passive Immune Transfer in Calves: A Meta-Analysis on the Consequences and Assessment of the Economic Impact
Source: PLoS One. 2016 Mar 17;11(3):e0150452. doi: 10.1371/journal.pone.0150452 (PMC4795751; doi:10.1371/journal.pone.0150452)
Supplement: S1 Fig — (PDF) [file pone.0150452.s001.pdf]

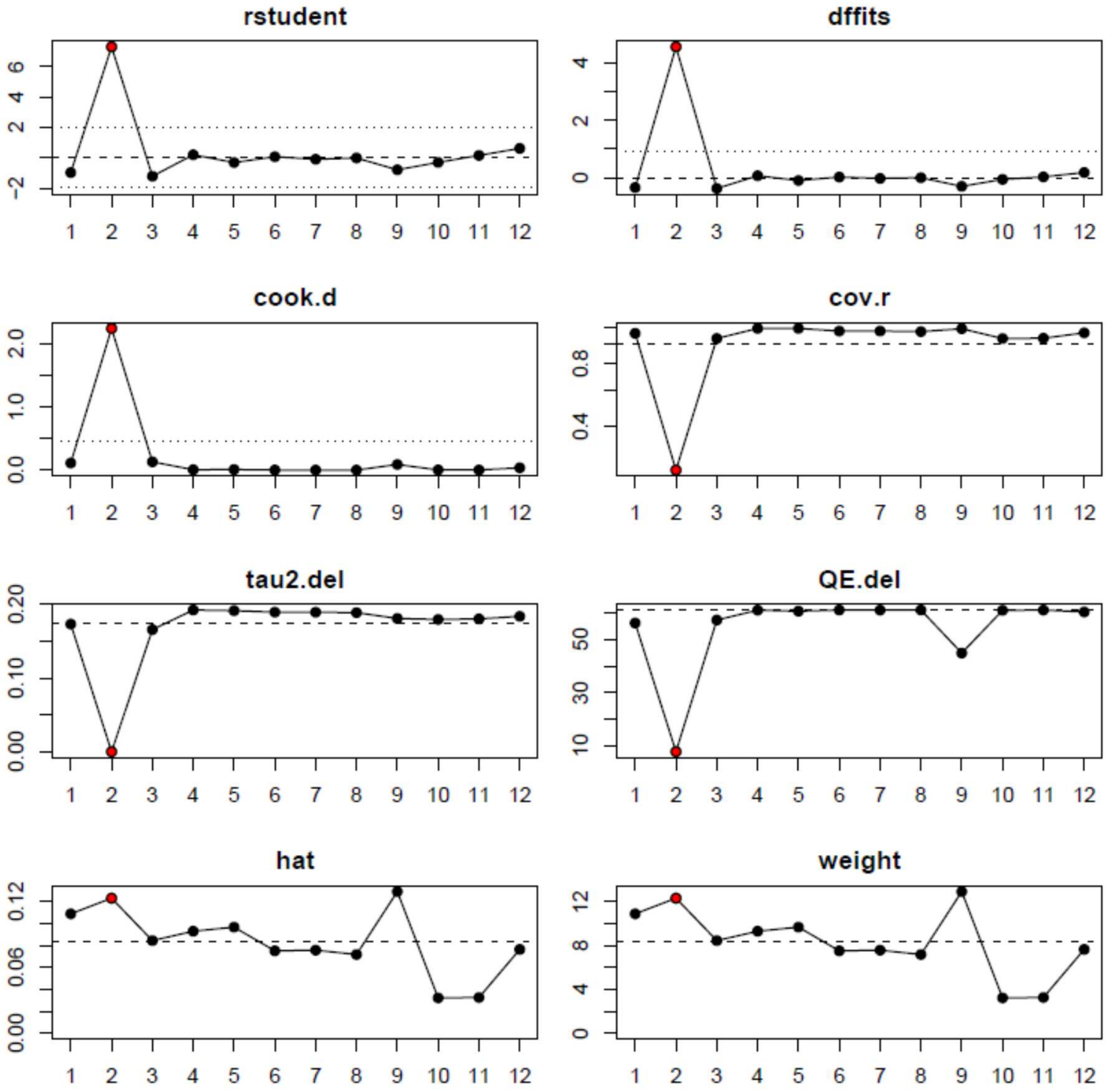

Figure S1. Example of an influential case diagnostics graph for respiratory diseases.

Plot of the externally standardized residuals, DFFITS values, Cook's distances,

covariance ratios, estimates of τ^2 and test statistics for (residual) heterogeneity when each

study is removed in turn, hat values, and weights for the 12 studies examining the risk of respiratory diseases in

cases of FPT.
