# Supplementary material for: Failure of Passive Immune Transfer in Calves: A Meta-Analysis on the Consequences and Assessment of the Economic Impact
Source: PLoS One. 2016 Mar 17;11(3):e0150452. doi: 10.1371/journal.pone.0150452 (PMC4795751; doi:10.1371/journal.pone.0150452)
Supplement: S1 Table — (PDF) [file pone.0150452.s002.pdf]

Table S1. Definitions of costs of diseases

	C_i (unit cost, €)			Ponderation coefficient			
	Low	Moderate	High	BRD	Diarrhoea	Omphalitis	Septicaemia
Antibiotic (parenteral route)	5	8.5	11	1	0.5	1	1
Antibiotic (local route)	0.5	1	2	0	1	0	0
No-steroid anti-inflammatory	5	8.8	14	1	0.5	0	1
Steroid anti-inflammatory		1.24		0.25	0	0	1
Solute for rehydration (oral route)	1.5	4.13	6.5	0	0.9	0	0
Solute for rehydration (intravenous route)	8	9.3	14	0	0.45	0	1
Veterinarian visit to farm	30	40	60	0	0.33	0	1
Omphalitis surgery	100	150	200	0	0	0.1	0
Cost of labour (15 min)		15.6		0.25	0.25	0	0
