# Supplementary material for: Failure of Passive Immune Transfer in Calves: A Meta-Analysis on the Consequences and Assessment of the Economic Impact
Source: PLoS One. 2016 Mar 17;11(3):e0150452. doi: 10.1371/journal.pone.0150452 (PMC4795751; doi:10.1371/journal.pone.0150452)
Supplement: S2 Table — (PDF) [file pone.0150452.s003.pdf]

Table S2. Raw data used for the meta-analysis and to calculate the prevalence of mortality in calves without failure of passive transfer

	Definition of FPT[1]				Risk (95% CI) for mortality in the case of FPT[1]				Covariates		
# of calves	Control (g/l)	FPT (g/l)	Prev[3]	Period	Prev[3]	Risk	Value	CI95%	U/M[4]	Breed[5]	References
2,278	TP ≥ 55	TP = 45-55	39	0-112	5.6	RR	1.32	0.944-1.85	U	D	[1]
2,149	TP ≥ 55	TP = 45-50	36	0-112	7.2	RR	2.21	1.63-3	U	D	[1]
1,740	TP ≥ 55	TP = 40-45	21	0-112	7.2	RR	3.1	2.23-4.32	U	D	[1]
1,434	TP ≥ 55	TP < 40	4	0-112	5.8	RR	4.64	2.77-7.77	U	D	[1]
3,479	TP ≥ 55	TP < 55	60	0-112	8.2	RR	2.05	1.58-2.67	U	D	[1]
1,000	IgG ≥12	IgG <12	28	0-180	4.3	RR	2.04	1.14-3.66	U	D	[2]
1,000	IgG ≥18	IgG <18	43	0-180	4.3	RR	2.17	1.18-3.97	U	D	[2]
246	IgG ≥5	IgG <5	13	0-100	6.1	RR	2.52	0.86-7.43	U	D	[3]
246	IgG ≥10	IgG <10	35	0-100	6.1	RR	1.24	0.46-3.37	U	D	[3]
246	IgG ≥15	IgG <15	na	0-100	6.1	RR	0.88	0.32-2.39	U	D	[3]
246	TP ≥ 45	TP < 45	na	0-100	6.1	RR	2.55	0.64-10.1	U	D	[3]
246	TP ≥ 50	TP < 50	27	0-100	6.1	RR	1.7	0.60-4.8	U	D	[3]
246	TP ≥ 55	TP < 55	48	0-100	6.1	RR	1.24	0.46-3.31	U	D	[3]
3,103	TP ≥ 54	TP < 54	na	0-30	na	RR	1.4	0.847-2.31	U	D	[4]
3,103	TP ≥ 54	TP < 54	na	0-180	11.7	RR	1.58	1.22-2.04	U	D	[4]
na	TP ≥ 54	TP < 50	na	0-180	11.7	RR	1.89	0.89-4.1	U	D	[4]
na	TP ≥ 54	TP = 50-54	na	0-180	11.7	RR	1.28	0.56-2.93	U	D	[4]
601	IgG ≥ 24	IgG < 24	33	0-90	na	OR	2.56	1.21-5.29	U	B	[5]
263	IgG ≥16	IgG < 8	33	0-163	3.0	OR	5.4	1.3-23.5	U	B	[6]
1,424	IgG ≥ 16	IgG < 8	14	0-200	2.7	OR	4.9	2.5-9.5	M[7]	B	[7]
1,338	IgG ≥ 16	IgG= 8-16	17	0-200	2.7	OR	1.2	0.3-4.1	M[7]	B	[7]
1,556	IgG ≥16	IgG < 16	23	0-200	2,7	OR	3,4	1,8-6,5	M[7]	B	[7]
1,556	IgG ≥ 24	IgG < 24	41	0-200	2,7	OR	2,7	1,34-5,36	M[7]	B	[7]
147	IgG ≥ 10	IgG < 10	44	0-56	16,3	OR	1,33	1,04-1,71	U	D	[8]
156	IgG ≥ 10	IgG < 10	19,05	0-28	40,38	HR[6]	26,2	7,6-90,38	M	D	[9]
156	IgG ≥ 10	IgG < 3.5	19	0-28	40,4	OR	35,5	7,92-160,77	U	D	[9]
221	IgG ≥ 10	IgG < 10	42,86	0-28	12,82	HR[6]	5,88	1,68-20,52	M	D	[9]

221	IgG ≥ 10	IgG = 3.5-10	43	0-28	12,8	OR	7,5	1,67-33,78	U	D	[9]
273	IgG ≥ 10	IgG < 10	62	0-28	21	OR	13,8	3,25-58,56	U	D	[9]
2,177	IgG ≥ 10	IgG < 10	41	0-56	5,7	RR	2,12	1,06-4,28	U	D	[10]

Na: not available. 1: Failure of passive transfer; 2: these animals are used as reference (no FPT) to calculate the OR. When not specified, they are the opposite of the definition of FPT; 3: Prevalence; 4: U=univariate model; M=multivariate model; 5: D=dairy; B=Beef; C: Crossed or undetermined. 6: HR were not included in the meta-regression. 7: Co-variates were dameline, sireline, and year.

References

1. Tyler JW, Hancock DD, Thorne JG, Gay CC, Gay JM. Partitioning the mortality risk associated with inadequate passive transfer of colostral immunoglobulins in dairy calves. J Vet Intern Med. 1999;13: 335-337.
2. Robison JD, Stott GH, DeNise SK. Effects of passive immunity on growth and survival in the dairy heifer. J Dairy Sci. 1988;71: 1283-1287.
3. Rea DE, Tyler JW, Hancock DD, Besser TE, Wilson L, Krytenberg DS, et al. Prediction of calf mortality by use of tests for passive transfer of colostral immunoglobulin. J Am Vet Med Assoc. 1996;208: 2047-2049.
4. Donovan GA, Dohoo IR, Montgomery DM, Bennett FL. Associations between passive immunity and morbidity and mortality in dairy heifers in Florida, USA. Prev Vet Med. 1998;34: 31-46.
5. Waldner CL, Rosengren LB. Factors associated with serum immunoglobulin levels in beef calves from Alberta and Saskatchewan and association between passive transfer and health outcomes. Can Vet J. 2009;50: 275-281.
6. Wittum TE, Perino LJ. Passive immune status at postpartum hour 24 and long-term health and performance of calves. Am J Vet Res. 1995;56: 1149-1154.
7. Dewell RD, Hungerford LL, Keen JE, Laegreid WW, Griffin DD, Rupp GP, et al. Association of neonatal serum immunoglobulin G1 concentration with health and performance in beef calves. J Am Vet Med Assoc. 2006;228: 914-921.
8. Priestley D, Bittar JH, Ibarbia L, Risco CA, Galvao KN. Effect of feeding maternal colostrum or plasma-derived or colostrum-derived colostrum replacer on passive transfer of immunity, health, and performance of preweaning heifer calves. J Dairy Sci. 2013;96: 3247-3256.
9. Berge AC, Besser TE, Moore DA, Sischo WM. Evaluation of the effects of oral colostrum supplementation during the first fourteen days on the health and performance of preweaned calves. J Dairy Sci. 2009;92: 286-295.
10. National Animal Health Monitoring System. Transfer of maternal immunity to calves. Washington, DC: National Dairy Heifer Evaluation Project (NDHEP), USDA; 1993.
11. Furman-Fratczak K, Rzasa A, Stefaniak T. The influence of colostral immunoglobulin concentration in heifer calves' serum on their health and growth. J Dairy Sci. 2011;94: 5536-5543.
12. Van Donkersgoed J, Ribble CS, Boyer LG, Townsend HG. Epidemiological study of enzootic pneumonia in dairy calves in Saskatchewan. Can J Vet Res. 1993;57: 247-254.

13. Virtala AM, Grohn YT, Mechor GD, Erb HN. The effect of maternally derived immunoglobulin G on the risk of respiratory disease in heifers during the first 3 months of life. Prev Vet Med. 1999;39: 25-37.

14. Windeyer MC, Leslie KE, Godden SM, Hodgins DC, Lissemore KD, LeBlanc SJ. Factors associated with morbidity, mortality, and growth of dairy heifer calves up to 3 months of age. Prev Vet Med. 2014;113: 231-240.

15. Virtala AM, Mechor GD, Grohn YT, Erb HN. Morbidity from nonrespiratory diseases and mortality in dairy heifers during the first three months of life. J Am Vet Med Assoc. 1996;208: 2043-2046.

16. Gow S, Waldner C, Ross C. The effect of treatment duration on weaning weights in a cow-calf herd with a protracted severe outbreak of diarrhea in calves. Can Vet J. 2005;46: 418-423, 425-416.
