# Supplementary material for: Failure of Passive Immune Transfer in Calves: A Meta-Analysis on the Consequences and Assessment of the Economic Impact
Source: PLoS One. 2016 Mar 17;11(3):e0150452. doi: 10.1371/journal.pone.0150452 (PMC4795751; doi:10.1371/journal.pone.0150452)
Supplement: S3 Table — (PDF) [file pone.0150452.s004.pdf]

Table S3. Raw data used for the meta-analysis and to calculate the prevalence of diseases in calves without failure of passive transfer

# of calves	Definition of FPT[1]			Risk (95% CI) for diseases in the case of FPT[1]						Covariates		References
	Control (g/l)	FPT (g/l)	Prev[3]	Disease	Period	Prev[3]	Risk	Value	95%CI	U/M[4]	Breed[5]	
175	IgG ≥ 5	IgG < 5	60	BRD, FD[6]	0-15	14	RR	1,44	0,94-2,19	U	D	[11]
175	IgG ≥ 10	IgG < 10	60	BRD, FD[6]	0-15	14	OR	5,79	4,35-7,69	U	D	[11]
325	IgG ≥ 8	IgG < 8	27	BRD, FD[6]	0-120	39	OR	1,205	0,69-2,53	U	D	[12]
325	IgG ≥ 8	IgG < 8	27	BRD, FD[6]	0-120	27	OR	2,44	1,38-4,3	U	D	[12]
375	IgG ≥ 12	IgG < 12	na	BRD, CD[6]	0-90	na	OR	1,9	1,1-3,2	M[12]	D	[13]
161	IgG ≥ 12	IgG < 12	na	BRD, CD[6]	0-90	na	OR	2,3	1,1-4,9	U	D	[13]
161	IgG ≥ 10	IgG < 10	na	BRD, CD[6]	0-90	na	OR	2,1	1,0-4,4	U	D	[13]
161	IgG ≥ 8	IgG < 8	na	BRD, CD[6]	0-90	na	OR	2,2	1,0-4,8	U	D	[13]
3300	TP ≥ 50	TP < 50	na	BRD, FD[6,9]	0-180	21	OR	1,27		U	D	[4]
3300	TP ≥ 50	TP < 50	na	BRD, FD[6,10]	0-180	21	OR	2,54		U	D	[4]
2874	TP ≥ 57	TP < 57	11	BRD, FD[6]	0-90	22	OR	1,6	1,3-2,0	M[13]	D	[14]
263	IgG ≥ 16	IgG < 16	31	BRD, FD[6]	0-162	47	RR	1,72	0,39-7,5	U	C	[6]
263	IgG ≥ 8	IgG < 8	25	BRD, FD[6]	0-162	47	RR	2,54	0,58-11,02	U	C	[6]
263	TP ≥ 48	TP < 48	19	BRD, FD[6]	0-162	47	RR	3,1	1,5-6,5	M[14]	C	[6]
175	IgG ≥ 5	IgG < 5	60	Dia[7]	0-160	36	RR	1,27	0,469-1,74	U	C	[11]
175	IgG ≥ 10	IgG < 10	60	Dia[7]	0-160	36	RR	1,32	0,978-1,78	U		[11]
175	IgG ≥ 15	IgG < 15	60	Dia[7]	0-160	36	RR	1,22	0,84-1,76	U		[11]
156	IgG ≥10	IgG ≤ 3.5	19	Dia-days, SD[7,11]	0-28	9	RR	2,88	2,22-3,71[18]	M		[9]
221	IgG ≥10	IgG =3.5-10	62	Dia-days, SD[7,] SD[7,11]	0-28	9	RR	1,85	1,5-2,8 [18]	M		[9]
263	IgG ≥16	IgG ≤ 8	22	all diseases	0-28	10	OR	6,4	0,4-10,7	M[15]	C	[6]
263	IgG ≥16	IgG = 8-16	11	all diseases	0-28	10	OR	2,1	2,6-15,7	M[15]	C	[6]
263	IgG ≥16	IgG ≤ 8	22	all diseases	0-163	20	OR	3,2	1,6-6,4	M[15]	C	[6]
263	IgG ≥16	IgG = 8-16	11	all diseases	0-163	20	OR	3,6	1,3-10	M[15]	C	[6]
1556	IgG ≥16	IgG ≤ 8	14	all diseases	0-200	12	RR	2,24	1,52-3,29	M[16]	D	[7]
1556	IgG ≥16	IgG = 8-16	55	all diseases	0-200	12	RR	1,38	0,80-2,39	M[16]	D	[7]
601	IgG ≥16	IgG ≤ 16	16	BRD, Dia, Omp[8]	0-90	18	OR	1,51	1,06-2,16	M[17]	D	[5]
147	IgG ≥10	IgG ≤ 10	55	BRD, Dia	0-56	61	RR	1,33	1,035-1,708	U	B	[8]

Na: not available. 1: Failure of passive transfer; 2: these animals are used as reference (no FPT) to calculate the OR. When not specified, they are the opposite of the definition of FPT; 3: Prevalence; 4 U=univariate model; M=multivariate model; 5: D=dairy; B=Beef; C: Crossed or undetermined. 6: Bovine respiratory diseases, diagnosed by farmer (FD), clinician (CD) or study personnel (SD). 7: Diarrhoea, or diarrhoea-days, diagnosed by study personnel (SD). 8: Omphalitis. 9: Winter, 2 days of treatment; 10: Summer, 2 days of treatment; 11: Definition of diarrhoea: watery and/or bloody faeces; 12: Covariates were Saison, Housing with adults and mastitis on cow; 13: Covariates were Saison, vaccine, previous disease and navel disinfection; 14: Covariate was sex; 15: Covariate was twin; 16: Covariates were year, pre-weaning, dameline, sireline and year; 17: Covariate was selenium status; 18: 90% CI.

References

1. Tyler JW, Hancock DD, Thorne JG, Gay CC, Gay JM. Partitioning the mortality risk associated with inadequate passive transfer of colostral immunoglobulins in dairy calves. J Vet Intern Med. 1999;13: 335-337.
2. Robison JD, Stott GH, DeNise SK. Effects of passive immunity on growth and survival in the dairy heifer. J Dairy Sci. 1988;71: 1283-1287.
3. Rea DE, Tyler JW, Hancock DD, Besser TE, Wilson L, Krytenberg DS, et al. Prediction of calf mortality by use of tests for passive transfer of colostral immunoglobulin. J Am Vet Med Assoc. 1996;208: 2047-2049.
4. Donovan GA, Dohoo IR, Montgomery DM, Bennett FL. Associations between passive immunity and morbidity and mortality in dairy heifers in Florida, USA. Prev Vet Med. 1998;34: 31-46.
5. Waldner CL, Rosengren LB. Factors associated with serum immunoglobulin levels in beef calves from Alberta and Saskatchewan and association between passive transfer and health outcomes. Can Vet J. 2009;50: 275-281.
6. Wittum TE, Perino LJ. Passive immune status at postpartum hour 24 and long-term health and performance of calves. Am J Vet Res. 1995;56: 1149-1154.
7. Dewell RD, Hungerford LL, Keen JE, Laegreid WW, Griffin DD, Rupp GP, et al. Association of neonatal serum immunoglobulin G1 concentration with health and performance in beef calves. J Am Vet Med Assoc. 2006;228: 914-921.
8. Priestley D, Bittar JH, Ibarbia L, Risco CA, Galvao KN. Effect of feeding maternal colostrum or plasma-derived or colostrum-derived colostrum replacer on passive transfer of immunity, health, and performance of preweaning heifer calves. J Dairy Sci. 2013;96: 3247-3256.
9. Berge AC, Besser TE, Moore DA, Sischo WM. Evaluation of the effects of oral colostrum supplementation during the first fourteen days on the health and performance of preweaned calves. J Dairy Sci. 2009;92: 286-295.
10. National Animal Health Monitoring System. Transfer of maternal immunity to calves. Washington, DC: National Dairy Heifer Evaluation Project (NDHEP), USDA; 1993.
11. Furman-Fratczak K, Rzasa A, Stefaniak T. The influence of colostral immunoglobulin concentration in heifer calves' serum on their health and growth. J Dairy Sci. 2011;94: 5536-5543.
12. Van Donkersgoed J, Ribble CS, Boyer LG, Townsend HG. Epidemiological study of enzootic pneumonia in dairy calves in Saskatchewan. Can J Vet Res. 1993;57: 247-254.

13. Virtala AM, Grohn YT, Mechor GD, Erb HN. The effect of maternally derived immunoglobulin G on the risk of respiratory disease in heifers during the first 3 months of life. Prev Vet Med. 1999;39: 25-37.

14. Windeyer MC, Leslie KE, Godden SM, Hodgins DC, Lissemore KD, LeBlanc SJ. Factors associated with morbidity, mortality, and growth of dairy heifer calves up to 3 months of age. Prev Vet Med. 2014;113: 231-240.

15. Virtala AM, Mechor GD, Grohn YT, Erb HN. Morbidity from nonrespiratory diseases and mortality in dairy heifers during the first three months of life. J Am Vet Med Assoc. 1996;208: 2043-2046.

16. Gow S, Waldner C, Ross C. The effect of treatment duration on weaning weights in a cow-calf herd with a protracted severe outbreak of diarrhea in calves. Can Vet J. 2005;46: 418-423, 425-416.
