# Supplementary material for: Failure of Passive Immune Transfer in Calves: A Meta-Analysis on the Consequences and Assessment of the Economic Impact
Source: PLoS One. 2016 Mar 17;11(3):e0150452. doi: 10.1371/journal.pone.0150452 (PMC4795751; doi:10.1371/journal.pone.0150452)
Supplement: S4 Table — (PDF) [file pone.0150452.s005.pdf]

Table S4. Raw data used for the review of ADG reduction in calves without failure of passive transfer

# of calves	Definition of FPT[1] or of disease			ADG decrease in case of FPT or disease					Covariates		References
	Control (g/l)	FPT (g/l)	Prev[3]	Period	ADG[3]	Value	P value	U/M[4]	Details	Breed	
1,511	IgG ≥ 22	IgG < 22	40	0-200	920	-16	0,05	M	Birth, sex, calving score	D	[7]
156	IgG ≥ 10	IgG < 3.5	19	0-60	1050	-120	0,01	M	Calving score, birth weight	D	[9]
221	IgG ≥ 10	IgG= 3.5-10	62	0-28	1050	-30	0,01	M	Calving score, birth weight	D	[9]
410	IgG ≥ 8	IgG < 8	na	0-30	394	-48	0,01	M	Season, treated pneumonia, age of dam	D	[13]
410	IgG ≥ 8	IgG < 8	na	0-90	565	-30[6]	0,01	M	Season, treated pneumonia, age of dam	D	[13]
1,000	IgG ≥ 12	IgG < 12	*28*	*0-35*	*285*	*-80*	*0,01*	M	Season of birth, age of dam	D	[2]
1,000	IgG ≥ 12	IgG < 12	*28*	*35-70*	*600*	*-160*	*0,01*	M	Season of birth, age of dam	D	[2]
1,000	IgG ≥ 12	IgG < 12	*28*	*70-105*	*628*	*-260*	*0,01*	M	Season of birth, age of dam	D	[2]
1,000	IgG ≥ 12	IgG < 12	*28*	*105-180*	*800*	*-90*	*0,01*	M	Season of birth, age of dam	D	[2]
1,000	IgG ≥ 12	IgG < 12	28	0-180	627	-134	0,01	M	Season of birth, age of dam	D	[2]
175	IgG ≥ 15	IgG < 15	5	365-AI1	640	-50	0,05	U		D	[11]
323	TP ≥ 52	TP < 52	11	0-90	950[7]	-8[7]	<0,01	M	Twin, age, vaccine, season of birth, main diseases	C	[14]
147	≥ 1 disease event		62	0-100	410	-89	0,001	U		D	[8]
263	Respiratory		47	W-242	860	-40	0,04	M	Weaning weight, Sex, Mastitis of dam	C	[6]
263	Morbidity		47	0-242	860	-66	0,01	M	Birth weight, weaning age, sex, type of birth	C	[6]
410	Respiratory[8]		na	0-90	565	-14[9]	0,01	M	Season, Body weight, Umbilical infection, Age of dam	C	[15]
321	Diarrhoea[9]		90	0-200	931	-76	0,0015	M	Sex	B	[16]
207	Diarrhoeal (trt days)		21	0-200	931	-28	0,04	M	Sex, age of dam	B	[16]

Na: not available. 1: Failure of passive transfer; 2: These animals are used as reference (no FPT) to calculate the OR. When not specified, they are the opposite of the definition of FPT; 3: Prevalence; 4 U=univariate model; M=multivariate model; 5: D=dairy; B=Beef; C: Crossed or undetermined; 6: Independent to pneumonia diagnosis. 7: Weight from heart-girth meaning tape; 8 Pneumonia duration (weeks) 9: Each week of pneumonia reduced the ADG by 9 g; 9: > 6 trt vs 0-1 trt.

References

1. Tyler JW, Hancock DD, Thorne JG, Gay CC, Gay JM. Partitioning the mortality risk associated with inadequate passive transfer of colostral immunoglobulins in dairy calves. J Vet Intern Med. 1999;13: 335-337.
2. Robison JD, Stott GH, DeNise SK. Effects of passive immunity on growth and survival in the dairy heifer. J Dairy Sci. 1988;71: 1283-1287.
3. Rea DE, Tyler JW, Hancock DD, Besser TE, Wilson L, Krytenberg DS, et al. Prediction of calf mortality by use of tests for passive transfer of colostral immunoglobulin. J Am Vet Med Assoc. 1996;208: 2047-2049.
4. Donovan GA, Dohoo IR, Montgomery DM, Bennett FL. Associations between passive immunity and morbidity and mortality in dairy heifers in Florida, USA. Prev Vet Med. 1998;34: 31-46.
5. Waldner CL, Rosengren LB. Factors associated with serum immunoglobulin levels in beef calves from Alberta and Saskatchewan and association between passive transfer and health outcomes. Can Vet J. 2009;50: 275-281.
6. Wittum TE, Perino LJ. Passive immune status at postpartum hour 24 and long-term health and performance of calves. Am J Vet Res. 1995;56: 1149-1154.
7. Dewell RD, Hungerford LL, Keen JE, Laegreid WW, Griffin DD, Rupp GP, et al. Association of neonatal serum immunoglobulin G1 concentration with health and performance in beef calves. J Am Vet Med Assoc. 2006;228: 914-921.
8. Priestley D, Bittar JH, Ibarbia L, Risco CA, Galvao KN. Effect of feeding maternal colostrum or plasma-derived or colostrum-derived colostrum replacer on passive transfer of immunity, health, and performance of preweaning heifer calves. J Dairy Sci. 2013;96: 3247-3256.
9. Berge AC, Besser TE, Moore DA, Sischo WM. Evaluation of the effects of oral colostrum supplementation during the first fourteen days on the health and performance of preweaned calves. J Dairy Sci. 2009;92: 286-295.
10. National Animal Health Monitoring System. Transfer of maternal immunity to calves. Washington, DC: National Dairy Heifer Evaluation Project (NDHEP), USDA; 1993.
11. Furman-Fratczak K, Rzasa A, Stefaniak T. The influence of colostral immunoglobulin concentration in heifer calves' serum on their health and growth. J Dairy Sci. 2011;94: 5536-5543.
12. Van Donkersgoed J, Ribble CS, Boyer LG, Townsend HG. Epidemiological study of enzootic pneumonia in dairy calves in Saskatchewan. Can J Vet Res. 1993;57: 247-254.

13. Virtala AM, Grohn YT, Mechor GD, Erb HN. The effect of maternally derived immunoglobulin G on the risk of respiratory disease in heifers during the first 3 months of life. Prev Vet Med. 1999;39: 25-37.

14. Windeyer MC, Leslie KE, Godden SM, Hodgins DC, Lissemore KD, LeBlanc SJ. Factors associated with morbidity, mortality, and growth of dairy heifer calves up to 3 months of age. Prev Vet Med. 2014;113: 231-240.

15. Virtala AM, Mechor GD, Grohn YT, Erb HN. Morbidity from nonrespiratory diseases and mortality in dairy heifers during the first three months of life. J Am Vet Med Assoc. 1996;208: 2043-2046.

16. Gow S, Waldner C, Ross C. The effect of treatment duration on weaning weights in a cow-calf herd with a protracted severe outbreak of diarrhea in calves. Can Vet J. 2005;46: 418-423, 425-416.
